# Using 3D modelling and printing to study avian cognition from different geometric dimensions

**DOI:** 10.1098/rsos.181938

**Published:** 2019-05-15

**Authors:** Canchao Yang, Wei Liang, Anders Pape Møller

**Affiliations:** 1 Ministry of Education Key Laboratory for Ecology of Tropical Islands, College of Life Sciences, Hainan Normal University, Haikou 571158, People's Republic of China; 2 Ecologie Systématique Evolution, Université Paris-Sud, CNRS, AgroParisTech, Université Paris-Saclay, F-91405 Orsay Cedex, France

**Keywords:** avian cognition, 3D modelling, 3D printing, stereoscopic structure, surface edges

## Abstract

Studying animal cognition is meaningful because it helps us understand how animals adapt to the natural environment. Many birds build nests, clean their nests and reject foreign objects from their nests, which provide an optimal opportunity for studying their cognition toward foreign objects in nests. However, hand-made models used in previous studies have many deficiencies that considerably constrain our capacity to understand the evolution of avian cognition of foreign objects because they are unquantifiable and dependent on different features. We established a 3D modelling and printing method to manipulate one geometric dimension of a model while controlling for others, which allowed us to investigate avian cognition for different dimensions independently. Here we introduce this method, conduct an empirical study as an example, and discuss its applications to further studies.

## 1. Introduction

Cognition refers to the mental capacities of animals responding to external or internal stimuli, behind which lies a series of physiological and neural activities that are determined by diverse genotypic traits combined with environmental factors [1,2]. Animal cognition determines how animals perceive and react to the natural environment, which further influences the fitness of animals [3–5]. For example, the class of birds has evolved cognition of objects in their nests, where they lay their eggs. Eggs contain offspring and parents take care of them prudently, thereby passing their genes successfully from one generation to the next [6]. Therefore, to provide optimal nursing conditions, like mammals including human beings [7], bird parents keep their nests clean and safe. Generally they recognize and get rid of all foreign objects falling into their nests, eggshells from hatching or damage, and faeces produced by nestlings (but see [8,9]). Such nest sanitation behaviour has evolved as a means to reduce the negative effects of parasites and pathogens on nestlings [10].

To clean their nests, bird parents need cognitive capacity to distinguish their eggs from other objects. Previous studies have used different shapes of models to study such cognition [9–16]. They found that nest sanitation is widespread in birds, and they recognize objects other than eggs with various frequencies depending on species and shapes of the object [17]. However, the mechanism behind the cognition of foreign objects, which is the key for explaining how birds distinguish eggs from other objects to avoid adoption of brood parasites, is still largely unknown because the models that have been used are hand-made and not quantifiably controlling for the associations among different shapes of models. For example, stick-shaped and coin-shaped models were generally used as non-egg-shaped models [13], although their shapes simultaneously change in different geometric dimensions. Even though birds recognize different shapes of models in different frequencies, the exact clues used for recognition of non-egg objects from eggs are uncertain. Although Igic *et al*. [18] has proposed the method of 3D printing to study avian cognition, their study refers to the method of establishing 3D egg-shaped models to replace hand-made models.

Here we established a method of 3D modelling and printing to create different shapes of models that are quantifiable with respect to different geometric dimensions (i.e. changing surface edge or stereoscopic structure by controllable parameters in the present study), and achieved to change models in one dimension while controlling for others (i.e. changing the surface edge or stereoscopic structure independently), which allowed us to test the exact clues for recognition. The aims of our study were to (i) introduce such a method and (ii) provide an experimental example by using this method in barn swallows (*Hirundo rustica*) that have been shown to possess nest sanitation capacities [13,14]. We predicted that the cognitive capacity of birds should increase after we manipulated the geometric dimension (i.e. the surface edge or stereoscopic structure of objects) from egg-shaped to non-egg-shaped properties. In other words, the higher the degree that objects deviate from egg-shaped properties, the higher the frequencies that the birds recognize these objects.

## 2. Material and methods

### 2.1. 3D modelling of surface edge

**Figure 1.**
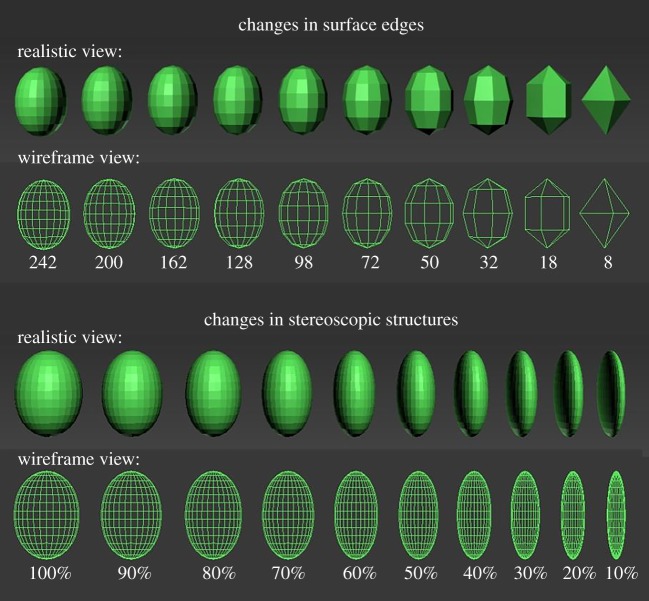
3D models with changes in surface edges and stereoscopic structure. Numbers on upper part refer to the number of flats on each model while percentages on lower part refer to the magnitude of reducing in stereoscopic structure.

**Figure 2.**
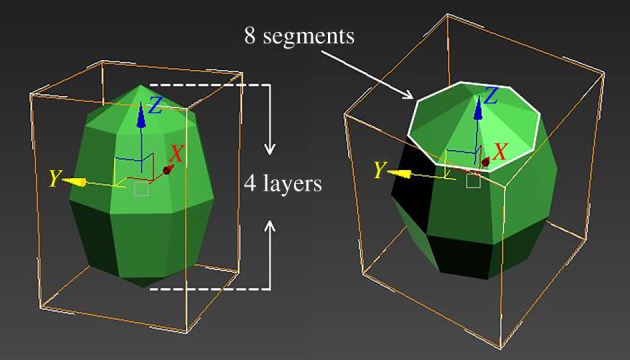
A 3D model with 32 flats on the surface that illustrates the segments and layers.

**Figure 3.**
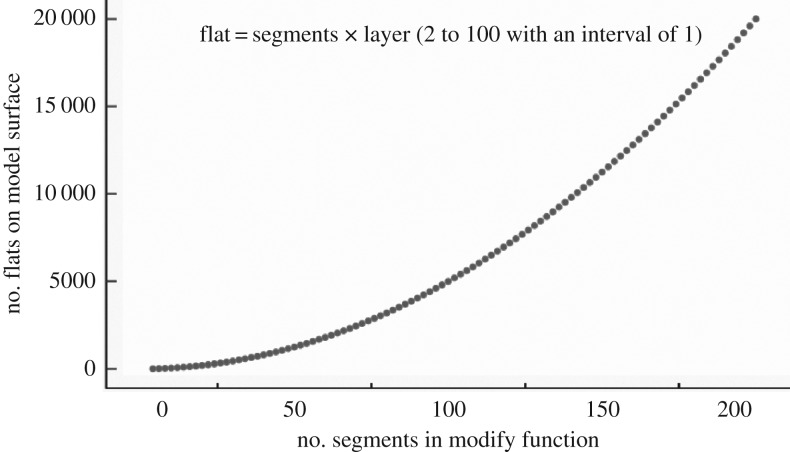
The relationship between the numbers of segments and flats on model surface.

In this study we manipulated two geometric dimensions of objects, called surface edges and stereoscopic structure, because they are obvious properties and easy to understand. In nature foreign objects falling into bird nests differ in their surface edges by some being round while others are sharper. For instance, foreign objects such as wild fruits are generally round in shape while stones are relatively sharper. Although these objects are likely to be removed, they change in different geometric dimensions that are hidden for cognition. To investigate the effect of surface edges in avian cognition, we should manipulate the geometric dimension of surface edges and control the effects of all other dimensions (the stereoscopic structure). Firstly, 3D Studio Max 2015 for Windows (Autodesk Inc., California, USA) was used to generate an elliptical model that represents an egg-shaped model. Then we used the modify function to change the segment parameter, which can create different degrees of surface edges (figure 1). The upper part of figure 1 presents 10 magnitudes of change in surface edges. The numbers refer to the number of flats (from 8 to 242) that compose the elliptical model, and they correspond to the number of segments (from 4 to 22 with an interval of 2) in the modify function. When an elliptical model was generated, it was composed of different layers, and the number of flats on each layer refers to the number of segments (figure 2). Thus the total number of flats on a model surface was the product of segments and layers. If we generated elliptical models with the number of segments from 4 to 200 with an interval of 2, the number of layers will be automatically produced from 2 to 100 with an interval of 1. Thus the number of flats changes from 8 to 20 000 with a cumulative increase (figures 1 and 3; table 1). The surface edges of a model became more and more rounded and undetectable with the increase in the number of flats on surfaces. By contrast, when the flats on surfaces decreased, the model became sharp and edged. Therefore, the changes in surface edges of models allow us to independently test the capacity and degree of edge detection in avian cognition.

**Table 1.** The relationship between model flats and segment in modify function.

segment	layer	flats	difference^a^
4	2	8	0
6	3	18	10
8	4	32	14
10	5	50	18
12	6	72	22
14	7	98	26
16	8	128	30
18	9	162	34
20	10	200	38
22	11	242	42
24	12	288	46
26	13	338	50
28	14	392	54
30	15	450	58
·	·	·	·
·	·	·	·
·	·	·	·
200	100	20 000	398

^a^Refers to the difference between two adjacent flats.

### 2.2. 3D modelling of stereoscopic structure

Here we generated another geometric dimension in models, the stereoscopic structure, which refers to the volume degree of objects in space. For example, plump objects such as wild fruit or stones generally have a higher degree of stereoscopic structure than leaves or small branches that are relatively pliable. To change the stereoscopic structure of a model while controlling for surface edges, we used the function of scale transformation in 3D Studio Max 2015 for Windows to compress one axis of the elliptical models while the other two axes and the number of flats on the surface was unaltered (figure 1). The lower part of figure 1 exhibits the changes in stereoscopic structure of an elliptical model that has a constant number of flats (450). The thickness (*y*-axis) of the model decreased 10% at each magnitude and thus it became more and more pliable (figures 1 and 2). If such a change in stereoscopic structure continues, finally the 3D model will become a 2D plane. Therefore, the changes in stereoscopic structure of a model allow us to test the capacity and degree of stereoscopic detection in avian cognition independently.

### 2.3. Models for example study

**Figure 4.**
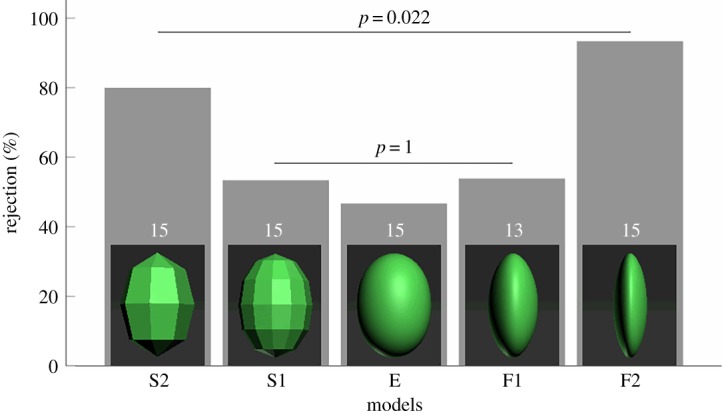
Rejection rates of different 3D models by barn swallows. Numbers in bars refer to sample sizes.

We generated an elliptical model (hereafter model E) with 20 000 flats on the surface and a size of 18.5 × 13.5 mm in length and width, respectively, which is similar to that of barn swallow eggs (mean ± s.d.: 18.2 ± 0.6 × 13.3 ± 0.5 mm, *n* = 12 clutches). Although such a size of models is slightly larger than swallow eggs, they would not affect rejection behaviour because our previous studies have indicated that barn swallows are grasp-rejecters that are capable of rejecting larger and heavier objects without rejection error [13,14]. Therefore, model E represents an egg-shaped model and it has a large number of flats of 20 000 that makes it as round as an egg surface so that its surface edge is undetectable. Then we modify and scale transform functions to change the surface edges and stereoscopic structure of model E, respectively. Two degrees of change were performed in each geometric dimension. The number of flats on model E was reduced to 72 (model S1) and further to 32 (model S2) as two magnitudes of change in surface edge (figure 4). In another geometric dimension, the thickness of model E was reduced to 9.5 mm (model F1) and further to 6 mm (model F2) as two magnitudes of change in stereoscopic structure while the surface edge was unaltered (figure 4). These five types of model were printed by a Digital Light Processing (DLP) 3D printer (StarRAY SP-DD230, Sprintray Inc., Guangdong, China) with photosensitive resin RD-203 (in green colour) as raw materials of models. We did not paint the models because (i) swallow eggs are maculate implying that painting with markings is hard to create the mimic egg pattern perfectly; (ii) hand-painting of egg markings may result in a risk of increasing variation among models, which may produce negative effect of cognition testing on geometric dimensions; and (iii) the intention of the experiment in this study focuses on the geometric dimension, and thus colour can be controlled by using the original colour of raw model materials. Additionally, we chose these five types of models (E, S1, S2, F1, F2) because they are obviously different and detectable in two geometric dimensions (i.e. changes from E to S1 and S2 for surface edges and changes from E to F1 and F2 for stereoscopic structure). This study intends to provide preliminary guidance for further studies, and thus it is inappropriate to choose models that are similar on surface edges or stereoscopic structure.

Barn swallow nests were searched for throughout the village Lingao (19°34′–20°02′ N, 109°3′–109°53′ E) located on Hainan Island, China. Each observed nest randomly received one of the five types of model on the day after clutch completion, and was then monitored for 6 days to confirm the response by the swallow nest owner, with the model classified as rejection if the model was ejected or deserted, or accepted if the model continued to be incubated [13]. To control for disturbance, we randomly selected *N* = 14 nests which were treated similarly as experimental nests except that no model was inserted.

Likelihood ratio test was used to compare the rejection rates among different models. IBM SPSS 20.0 for Windows (IBM Inc., USA) was used to achieve the analyses. All tests were two-tailed and the significant level was *p* < 0.05.

## 3. Results

In this example study, barn swallows rejected about half of model E (46.7%, *n* = 15), and the rejection rates increased to 53.3% (*n* = 15) in model S1 and to 80% (*n* = 15) in model S2 (figure 4). For model F1 to F2, the rejection rates were 53.8% (*n* = 13) to 93.3%, respectively. All rejection events were executed by ejection. The rejection rates were significantly different among all five models (*χ*^2^ = 12.068, d.f. = 4, *p* = 0.022, likelihood ratio test), but not among models E, S1 and F1 (*χ*^2^ = 0.187, d.f. = 2, *p* = 0.1, likelihood ratio test).

## 4. Discussion

By using models that are manipulated one geometric dimension at a time, while controlling for the other, (i) the barn swallow was found to distinguish non-egg-shaped from egg-shaped objects by both surface edges and stereoscopic structure, elucidating that these two clues for detection have both evolved in swallows; (ii) consistent with our prediction, the cognitive capacity of swallows on both surface edges and stereoscopic structure increased with the changes in deviation from egg-shaped to non-egg-shaped properties; (iii) the tendencies of rejection toward these two geometric dimensions (i.e. surface edges and stereoscopic structure) were consistent so that they both increased slightly after the first but sharply after the second magnitude of changes in such properties, implying that the evolution of these two clues for detection in swallows may be a case of parallel evolution; and (iv) swallows were considerably more sensitive to the second magnitude of change in both geometric dimensions, providing information on the cognitive precision on surface edge and stereoscopic structure in swallows. If more degrees of model were used, the precision might even be more distinct.

In summary, using 3D modelling and printing (see also [18,19]) to generate models in different geometric dimensions has several advantages compared to previous methods: (i) models are more consistent and quantifiable; (ii) we can manipulate one geometric dimension of models while controlling for others, which allows to test the avian response to one clue independently; (iii) independently testing different geometric dimensions allows us to know the evolutionary circumstances in detection of different dimensions in birds; (iv) by manipulating two or more geometric dimensions but controlling for others, we can also investigate the effect of interaction between or among different dimensions on avian cognition; and (v) by using a sufficient number of models that change gradually in geometric dimension, we can get information on cognitive precision of the studied object and compare it with other populations or species. It is necessary to mention that this study used two geometric dimensions, the surface edges and stereoscopic structure, which are obviously detected by human eyes. There should be more geometric dimensions [20] that are waiting for discovery. Our study provides preliminary guidance, and we suggest that ornithologists explore more geometric dimensions of models by using this method that would help us better understand the evolution of cognition in birds. Moreover, here we used the original colour of the resin raw material for 3D printing. We suggested that further studies paint the models with different colours to investigate the interaction between colours and geometric dimensions on avian cognition.

## Data accessibility

The dataset supporting this article can be found in the Dryad Digital Repository: https://doi.org/10.5061/dryad.45bp07g [21].
